# The transversoclasiotome: a novel instrument for examining the vertebral artery

**DOI:** 10.1007/s12024-023-00638-x

**Published:** 2023-05-24

**Authors:** Rafael Boscolo-Berto, Veronica Macchi, R. Shane Tubbs, Aron Emmi, Carla Stecco, Marios Loukas, Andrea Porzionato, Raffaele De Caro

**Affiliations:** 1 Institute of Human Anatomy, Department of Neurosciences, University of Padova, Via A. Gabelli 65, 35127 Padua, Italy; 2 Veneto Region Reference Center for the Preservation and Use of Gifted Bodies, Veneto Region, Padua, Italy; 3 National Reference Center for the Preservation and Use of Gifted Bodies, Padua, Italy; 4 Department of Neurosurgery, Tulane Center for Clinical Neurosciences, Tulane University School of Medicine, New Orleans, LA USA; 5 Department of Neurology, Tulane Center for Clinical Neurosciences, Tulane University School of Medicine, New Orleans, LA USA; 6 Department of Neurosurgery and Ochsner Neuroscience Institute, Ochsner Health System, New Orleans, LA USA; 7 Department of Anatomical Sciences, St. George’s University, St. George’s, Grenada

**Keywords:** Body Donation Program, Cadaver lab, Foramina transversaria, Transversoclasiotome, Vertebral artery, Patent

## Abstract

Opening the foramen transversarium of the cervical vertebrae is necessary for accessing the vertebral vessels. There are no specialist tools for cutting the anterior lamina of the transverse processes, and alternatives lead to questionable results. A novel tool, the transversoclasiotome, is described and tested. The literature and patent databases were systematically reviewed. A blueprint of the transversoclasiotome was created, and the prototype was tested through autopsy on ten fresh-frozen cadavers within our Body Donation Program. The transversoclasiotome consists of two delicate branches mounted as a scissor, one a cutting jaw and the other a knocker with a rounded tip, both angled 30° to the principal axis. The jaws shut, facing each other in parallel. The cutting jaw corresponds to a slit on the knocker profile without protruding beyond it even when entirely closed. It acts by cutting and wedging. The testing autopsies demonstrated its suitability for its purpose, with an adequate response to the pressure exerted on the bone lamina. The section cut cleanly, without sliding off while closing on the bone. The vertebral vessels were not injured either during instrument insertion or cutting. Their morphological features are described. The transversoclasiotome has been proven appropriate for sectioning the anterior lamina of transverse processes of the cervical vertebrae. It meets the needs of clinical anatomy in teaching and training clinicians or surgeons, forensic clinical anatomy during medico-legal investigation, and research.

## Introduction

The practical study of human anatomy is a central part of healthcare education and experimental research, from academic pre-graduation teaching to continuing medical education and training. It has widely influenced modern medicine’s development and scientific progress and still affects it profoundly [1–4]. Practical expedients such as virtual simulations or dummies have been introduced to complement the teaching of human anatomy, mainly to address the shortage of cadavers and anatomical parts from body donation programs and surgical activity [5–9]. However, despite the impressive progress of computer technology, anatomical dissection cannot be wholly replaced by any surrogate. In recent years, there has been a renewed interest in dissection, with the spread of body donation centers and the standardization of the requirements necessary for starting activities on the bodies so that it becomes a routine activity in universities [10].

This trend goes hand in hand with the increasing interest among medical disciplines, such as neurosurgery, vascular surgery, and orthopedics, in cadaver practice, the so-called cadaver lab, to test acquired knowledge, learn new surgical techniques, and train in operative procedures [11–14]. Specifically, anatomical dissection of the cervical region to access the spinal cord and neck vessels is of utmost interest for anatomy teaching, neuroanatomy research, surgical training, and medico-legal purposes. This is in line with the ongoing international expansion of the bio-medico-legal sciences, particularly forensic pathology and clinical forensic medicine, of which medical malpractice and the analysis of injury mechanisms are emerging topics in devoted scientific journals [15–17].

In this framework, the role of forensic clinical anatomy as a further evolution of clinical anatomy from a medico-legal perspective has long been recognized and integrated into the European guidelines for medico-legal investigation in cases of alleged medical malpractice [18, 19]. Notably, anatomical dissection of the cervical region is recommended for assessing neurological and vascular diseases that could affect the central nervous system [20, 21], providing detailed morphological descriptions of the subtended alterations [22]. In this setting, partial sectioning of the transverse process of the cervical vertebrae is necessary for gaining access to the foramen transversarium with exposure of the vertebral vessels.

However, as no dissection instruments are designed for this specific purpose, devices designed for other tasks are generally used, with questionable results regarding effectiveness, precision, and accuracy. Consequently, the dissection’s experimental, educational, and forensic value can be prejudiced, along with the integrity of the morphological data gathered. This is relevant in the medico-legal field, where careful anatomical dissection enables morphologically preserved samples to be collected. Otherwise, there could be interpretative errors based on biased premises with professional, deontological, ethical, and moral implications.

In this paper, the transversoclasiotome is described. It is a novel dissection instrument that facilitates the isolation of vertebral vessels, as demonstrated by practical application to cadavers.

## Material and methods

### Literature and patent searches

In March 2021, the literature was systematically reviewed by searching online databases to identify publications concerning any surgical or dissection instrument for isolating vertebral vessels. Medline/PubMed, Web of Science, Ovid, and Scopus were searched through a complex query, which included “free-text” combinations joining the terms “((vertebral AND (vessel* OR arter* OR vein*)) AND (isolat* OR dissect* OR section*) OR (vertebr* OR rachid*)) AND ((surgic* OR dissect*) AND (instrument* OR tool* OR device*))” into full text, as described previously [23]. Broad search terms were used with no temporal limits or language restrictions to ensure that relevant studies were not overlooked. Additional references from the papers included were checked for pertinent information.

Also, national and international patent databases (World Intellectual Property Organization (WIPO), European Patent Office, Ufficio Italiano Brevetti e Marchi (UIBM), Google Patents) were reviewed for surgical or dissection devices resembling the instrument described above.

### Technical drawing

For technical drawing and 3D rendering of the instrument, the transversoclasiotome, dedicated software was used (FreeCAD v0.18.4, as available at https://www.freecadweb.org/index.php), and files were created in.dwg format.

### Application to anatomical dissection

The transversoclasiotome was used to open the transverse foramina in ten unembalmed cadavers. Subjects aged 65–88 (mean age 77, four males and six females) were included in the study. Exclusion criteria were a history of neck trauma, gross evidence of congenital or acquired vertebral disease, and previous cervical spine surgery. All the procedures were performed on human bodies from the Body Donation Program “Donation to Science” of the University of Padova [24] and Veneto Region/National Reference Center for preserving and using gifted bodies (Deliberation of the Regional Council of the Veneto Region n. 245, March 8th, 2019; n. 389,897), in accordance with national laws and the ethical standards of the regional/national research committees, and with the 1964 Helsinki Declaration and its later amendments or comparable ethical standards. Written informed consent was provided to join the Body Donation Program. The privacy rights of human subjects were consistently observed. The cadavers were dissected anatomically in the supine position. The skin incision followed the anterior midline from between the mental protuberance to the jugular notch, and the anatomical dissection progressed layer-by-layer on both sides, including fasciae and muscles.

The platysma was identified in the subcutaneous tissue and sectioned vertically. The sternocleidomastoid muscle was detached at its insertions and removed, along with the infrahyoid muscles (sternohyoid, sternothyroid, thyrohyoid, and omohyoid). The carotid sheath was opened, and the internal jugular vein and the vagus nerve were sectioned and removed, but the common carotid artery was preserved. Once the triangle of the vertebral artery was recognized, delimited by the lateral edge of the longus colli muscle, the medial edge of the anterior scalene muscle, and the first part of the subclavian artery, the anterior scalene and longus colli muscles were detached and removed to reveal the caudal segments of the vertebral vessels. The origin of the vertebral artery from the subclavian artery was identified, and so was the connection of the vertebral vein to the brachiocephalic vein. The longus capitis and the rectus capitis anterior muscles were detached and removed. The cervical and brachial plexuses were identified. The anterior lamina of the transverse process of the cervical vertebrae was removed through two sagittal sections tangential to the medial and lateral contours of the foramen transversarium; both were made using the transversoclasiotome. The intertransverse muscles were detached and removed. The procedure was repeated from C6 to C1, and the contents of the foramina transversaria were examined.

## Results

In the literature and among the patents in the queried databases, no dedicated surgical or dissection instruments were identified to facilitate the isolation of vertebral vessels. Several tools such as chisels, osteotomes, bone knives, forceps, ossivorous pliers, and rachiotomes have been adapted for interventions in the spine but without special details to allow their specific and targeted use on the laminae of the transverse processes of the cervical vertebrae (Table 1).

**Table 1:** Results obtained by consulting the online patent databases regarding surgical or dissection devices resembling the transversoclasiotome being designed

Patent number	Title
WO2018040918A1	Ultrasonic osteotome tool bit
CN204971449U	Thin slice formula pedicle of vertebral arch osteotome
CN205054368	Arc crew cut vertebral plate osteotome that decorticates
CN204072218	Lumbar vertebrae undermining decompression osteotome
CN203369939U	Minimally invasive vertebral pedicle osteotome
CN203029345U	Depth-limiting osteotome for lower lumbar vertebral plates
CN202821534U	Extruded type spinal vertebral body osteotome
CN201211214Y	Vertebrae osteotome
CN201168021Y	Medical arcuated osteotome head
CN201168008Y	Bone knife for cutting vertebral pedicle
CN101283920	Abnormal undercut osteotome with bifurcated frontend
CN201123841Y	Medical slanting arc edge osteotome head
US5722977A	Method and means for anterior lumbar exact cut with quadrilateral osteotome and precision guide/spacer
CN2527233	Depth-limiting vertebral lamina osteotome
US00D324424S	Spinal osteotome

**Fig. 1:**
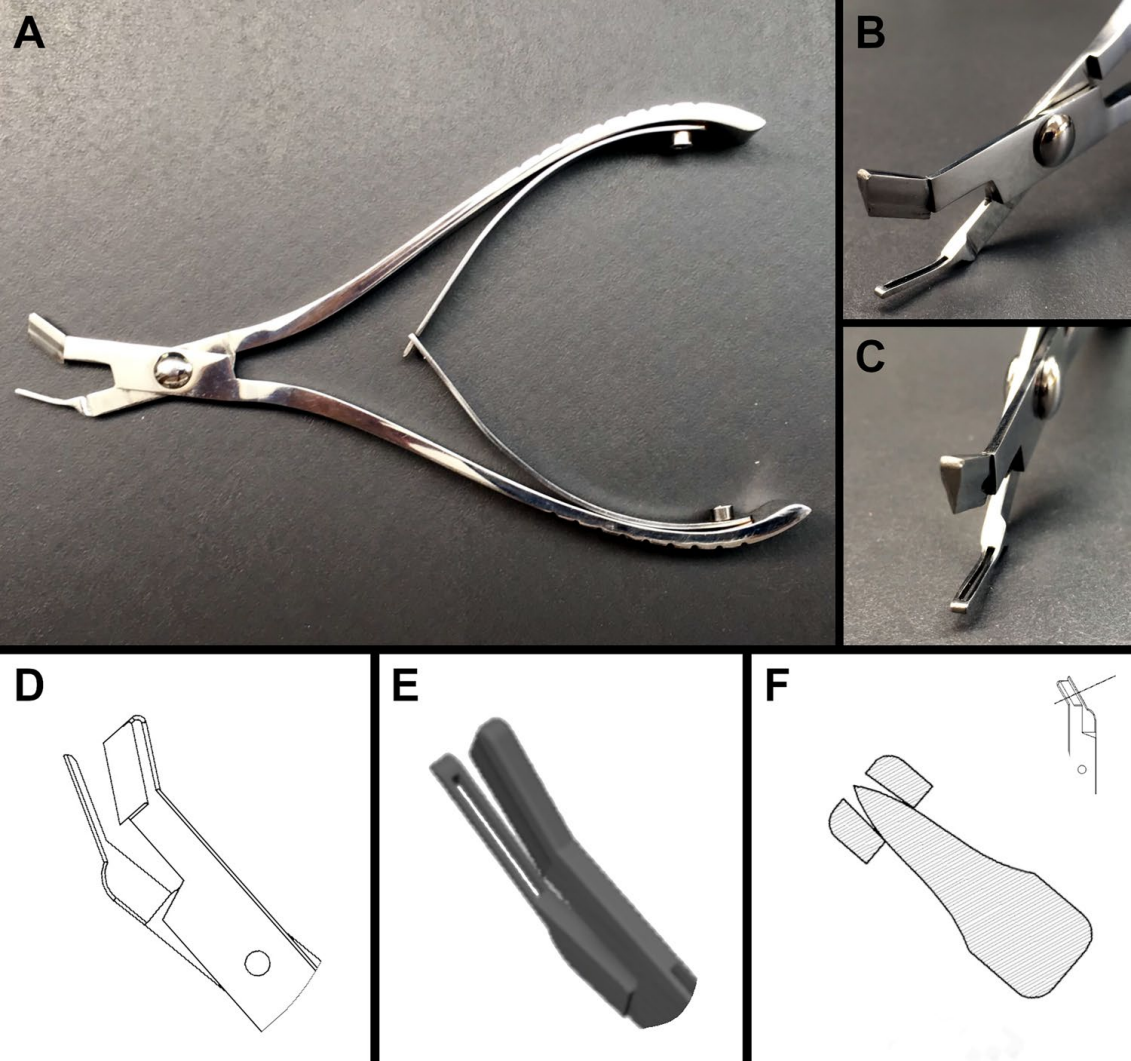
Technical drawing and 3D rendering of the transversoclasiotome. **A** Transversoclasiotome. Lateral view. Note the two delicate branches mounted as a scissor, one a cutting jaw and the other a knocker with a rounded tip, both angled at 30° to the principal axis. Moreover, a metal arch system is provided between the handpieces of the branches to facilitate the return of the jaws to the opening position after cutting. **B** Transversoclasiotome. Anterior oblique view. Note that the cutting jaw corresponds to a slit on the knocker profile. **C** Transversoclasiotome. Anterior oblique view. The two side surfaces of the cutting jaw, which converge to form the cutting edge, have a slight external convexity. Note the slit on the knocker profile. **D** Technical drawing. Lateral view. Note the two delicate branches mounted as a scissor, one a cutting jaw and the other a knocker with a rounded tip. The jaws (here partially opened) shut facing each other in parallel. This ensures cleanly cut sectioning, without the transversoclasiotome sliding off while closing on the bone. **E** 3D rendering. Oblique view from above. Note that the cutting jaw corresponds to a slit on the knocker profile. **F** Technical drawing. Cross-section of the jaws in complete closure as indicated in the scheme on the top right. Note that the cutting jaw corresponds to a slit on the knocker profile, without protruding beyond it even if entirely closed. The two side surfaces of the cutting jaw, which converge to form the cutting edge, have a slight external convexity. This favors a shift of the bone margins with a “wedge” mechanism during the mechanical sectioning

Therefore, a specialized dissection instrument with innovative solutions to the challenges of use on the cervical spine was developed (Fig. 1). The invention, named the transversoclasiotome, is a tool intended for manipulation by the anatomist/surgeon. It consists of two branches made mutually integral by a suitably tightened mounting screw to allow residual mobility in the reciprocal opening/closing of the jaws. One branch has a cutting end (the upper one), and the other a knocker (the lower one). The cutting edge closes on the knocker to dissect the bone lamina between them.

The main features of the transversoclasiotome are as follows (Fig. 1):

- The scissor shape allows the cutting energy to be transferred gradually during the execution of the technique.
- The extreme delicacy of the instrument in cross-section, especially in the jaws, allows its terminal part to be introduced into the slender foramen transversarium of the cervical vertebrae, occupied by blood vessels.
- The jaws form a terminal angle of 30° with the principal axes of the tool, which makes the transversoclasiotome ergonomic, since the cadaver/patient is placed in a supine position and the anatomist/surgeon is upright at the side.
- The knocker profile has a slit corresponding to the cutting edge on the opposite jaw to reduce wear on the blade. The cutting edge does not protrude beyond the external/lower profile of the knocker, so the vessels occupying the foramen transversarium of the cervical vertebrae are not injured.
- The two side surfaces of the cutting jaw, which converge to form the cutting edge, have a slight external convexity. This favors the shift of the bone margins with a “wedge” mechanism during the mechanical sectioning, thus promoting the cutting action and reducing the force required to obtain complete instrument closure.
- The closing system of the jaws ensures that they shut facing each other almost in parallel (“guillotine”) instead of a more conventional “scissor” closure. This limits the sliding of the instrument while the bone lamina is being sectioned and precludes escape of the lamina at the front of the instrument.
- The tip of the knocker jaw is rounded to prevent injury to the vertebral vessels in the foramen transversarium of the cervical vertebrae.
- A generic metal arch system is provided between the handpieces of the branches, to facilitate the return of the jaws to the opening after cutting.

**Fig. 2:**
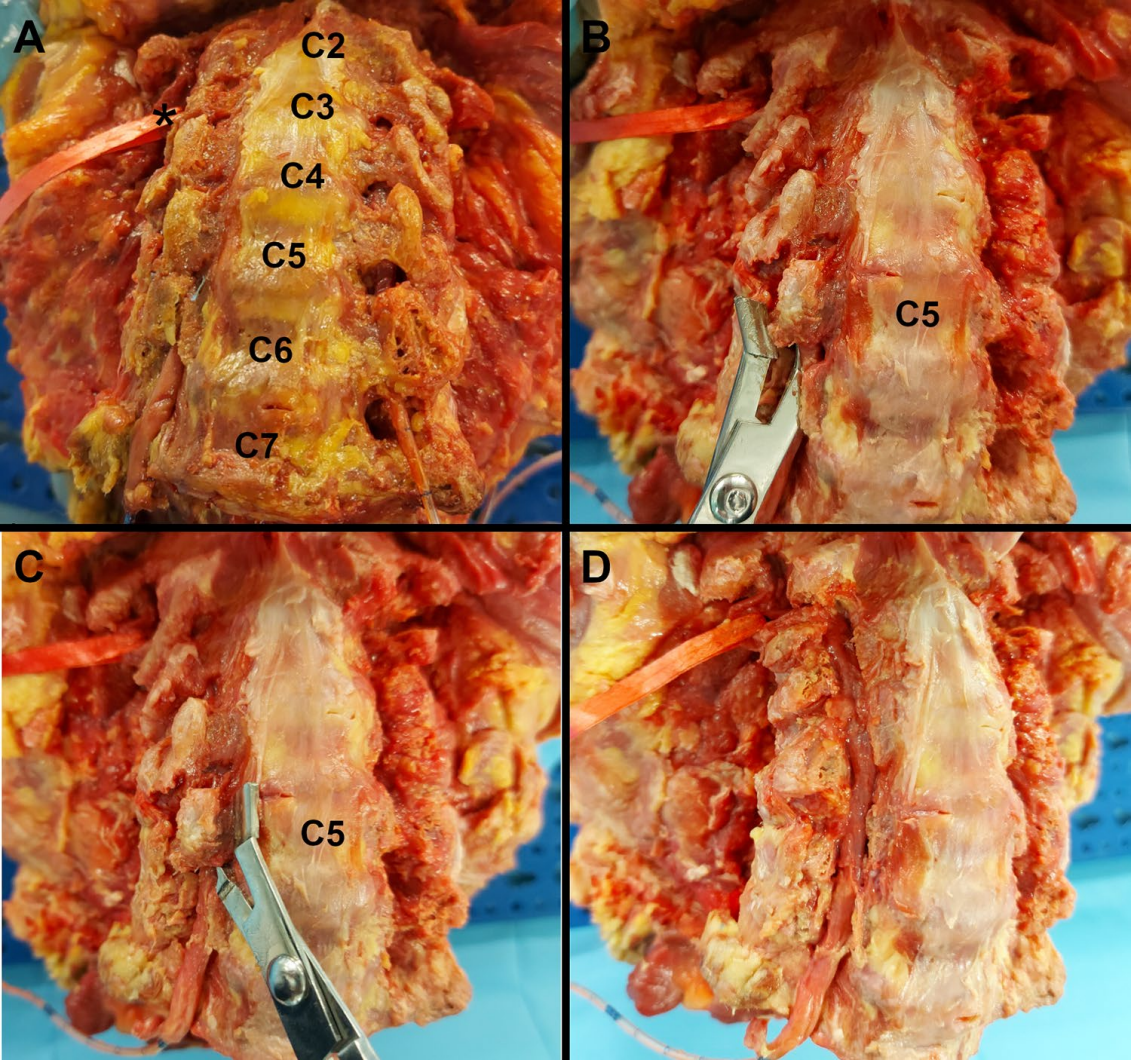
Bone plane of a craniocervical complex. Anterior view. How to use the transversoclasiotome. **A** Note the entry of the right and left vertebral arteries into the foramina transversaria starting at C6 and up. On the right side, the cranial loop of the V2 segment of the vertebral artery was suspended with a red ribbon (*). **B** A sagittal section tangential to the lateral contour of the foramen transversarium was performed to allow the removal of the anterior lamina of the transverse process of the cervical vertebra (C5). The procedure was previously completed using the transversoclasiotome for the anterior lamina of the transverse process of the C6 on the right, and for all the transverse processes of the left side with the harvesting of the vertebral artery. **C** A sagittal section tangential to the medial contour of the foramen transversarium was performed to allow the removal of the anterior lamina of the transverse process of the cervical vertebra (C5). The procedure was previously completed using the transversoclasiotome for the anterior lamina of the transverse process of the C6 on the right, and for all the transverse processes of the left side with the harvesting of the vertebral artery. **D** By repeating for C4 to C1 vertebrae what showed in **B** and **C** for the C5 (and previously C6), the complete exposure of the segment V2 of the vertebral artery is easily obtained, allowing its harvesting as shown for the left side

**Fig. 3:**
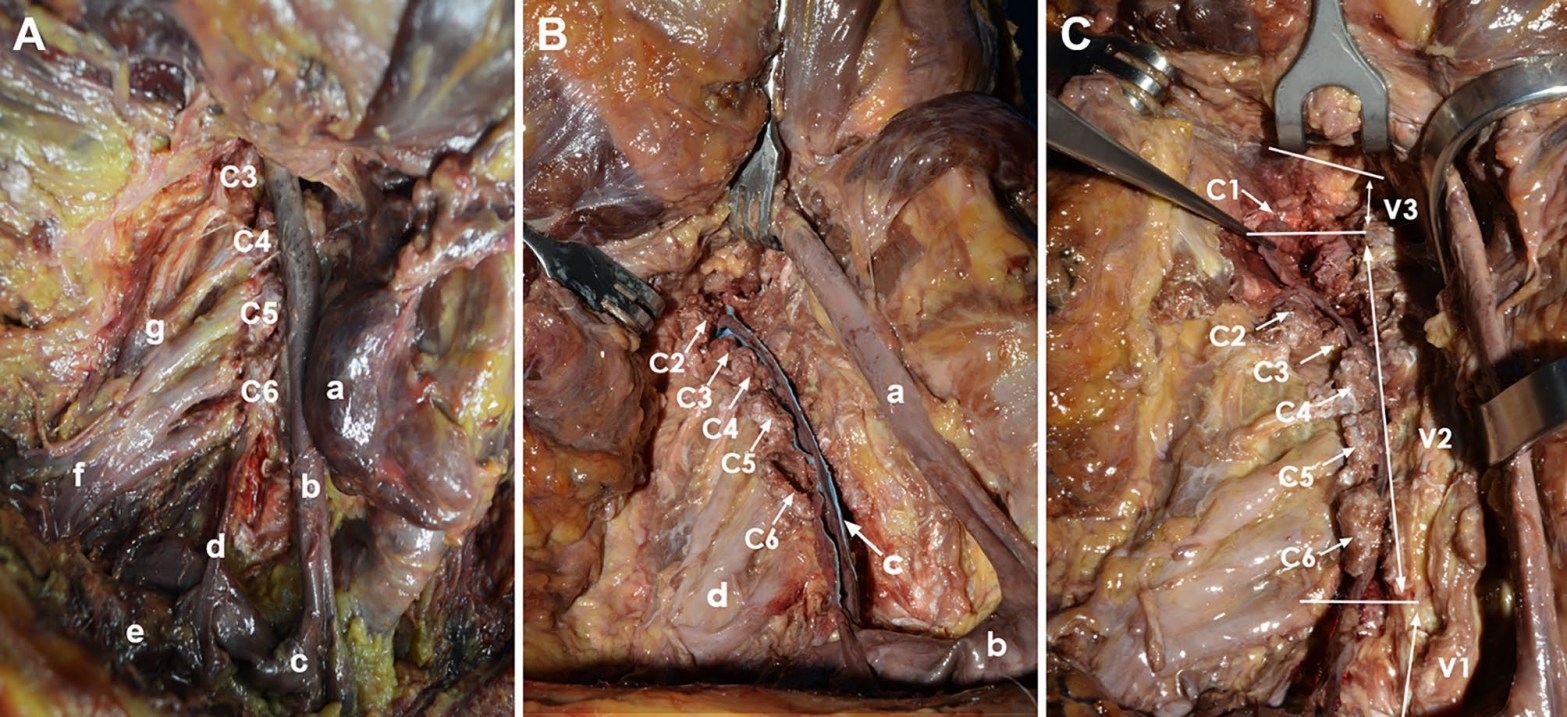
Anterior region of the neck between the middle and the deep cervical planes. Anterior view. **A** Dissection of the cervical region. a, right lobe of the thyroid gland; b, common carotid artery; c, subclavian artery; d, vertebral vein; e, subclavian vein; f, brachial plexus; g, cervical plexus. C3–C6, cervical vertebrae from C3 to C6. **B** Dissection of the cervical region. The anterior lamina of the transverse processes of cervical vertebrae C2–C6 and the intertransverse muscles were sectioned and removed to expose the vertebral artery. The vertebral artery (c) has been highlighted with blue marker. a, common carotid artery; b, subclavian artery; c, vertebral artery; d, brachial plexus; C2–C6, cervical vertebrae from C2 to C6. **C** Dissection of the cervical region. The anterior lamina of the transverse processes of cervical vertebrae C1–C6 were sectioned and removed. The course of the vertebral artery and its surgical partitioning are shown. V1, ostial or preforaminal segment; V2, foraminal segment; V3, suboccipital or extradural or extraspinal segment. The foraminal segment (V2) ascends through the foramina transversaria of the cervical vertebrae from C6 to C1, anteriorly to the roots of cervical plexus. The intertransverse muscles have been removed to expose the vertebral artery

The foramen transversarium of the cervical vertebrae was quickly opened during anatomical dissection, moving the transversoclasiotome upwards from C6 to C1 (Fig. 2). The vertebral vessels contained therein were identified, without documenting dissection artifacts due to the opening of the foramina transversaria. The vertebral artery was isolated from its origin to the foramen magnum. Similarly, the vertebral vein was isolated up to its connection with the brachiocephalic vein. The following findings were noteworthy (Fig. 3):

- The anterior lamina of the transverse process of the cervical vertebrae was approximately one-third of the posterior one.
- The vertebral artery was accompanied by a venous plexus, which in the caudal part converged into one vessel before its connection to the brachiocephalic vein once it had emerged from the foramen transversarium of the sixth cervical vertebra.
- In their course between the neighboring foramina transversaria, the vertebral vessels were contained within a musculoskeletal casing formed by intertransverse muscles inserted into the transverse processes.
- The vertebral artery was enclosed in a periosteal sheath continuing the one that covered the foramina transversaria, within which it was free to slide only a little. The periosteal sheath formed a proximal fibrous ring at the entrance of the vertebral artery into the foramen transversarium of C6. Likewise, at some points, the vertebral artery adhered to the periosteum, from which it had to be detached.
- The vertebral artery gave rise to small spinal branches passing through the intervertebral foramina to enter the vertebral foramen, muscular branches at the C1 level, and the posterior spinal artery just before it perforated the dura mater.
- The transverse process of C2 was inclined downwards, leading to an almost sagittal arrangement of the foramen transversarium, unlike all the other cervical vertebrae in which it was horizontal.
- The upper side of the posterior vertebral arch just behind the lateral mass of the atlas was directly reached once the anterior lamina of the transverse process of C1 was sectioned.

## Discussion

The anterior anatomical and surgical approach to the vertebral vessels is of interest for clinical and surgical teaching to undergraduate students, graduates, and surgical residents devoted to the craniocervical region; for medico-legal experts and practitioners of forensic clinical anatomy; and for scientific research [14, 18].

In the forensic field, there are many areas of application, generally aimed at the analysis of injury mechanisms, such as traumatic vertebral artery injuries due to penetrating (i.e., firearm or stab wound related) or nonpenetrating trauma (i.e., blunt force, hyperextension and rotation of the neck) [17, 25–27]. Several studies emphasize the clinical relevance of the vertebral artery and the opportunity to routinely examine it in all cases of fatal traumatic head and neck injuries [26, 28–30]. This is true for adults and even more so for infants and young children. For this latter, the neck is supported by a weaker musculoskeletal system and is therefore exposed to whiplash injury for sudden extension-and-flexion movement, as reported in shaken baby syndrome [31].

In adults, traumatic vertebral artery injury may be due to motor vehicle accidents, related to seat belt injury or assault with/without intent to kill [32–34]. In those settings, a subarachnoid hemorrhage could result from the trauma, or a misdiagnosed delayed laceration/dissection of the vertebral artery could lead to death, which can, in turn, raise issues of medical malpractice [35]. The precipitating event could be a minor trauma such as hyperextension or sudden rotation, causing a lesion of the vertebral artery [36]. This sometimes occurs during surgical and anesthesia procedures or chiropractic manipulation, raising hypotheses of medical malpractice [37–39]. The trauma could occur during sports participation, such as rugby, ice hockey, martial arts, golf, and running. It is due to specific mechanical stresses on the vertebral artery, which could be potentially fatal to the sportsman [40].

However, its examination is often neglected because of the difficulty of accessing by dissection procedure to the vertebral arteries, and no workarounds are available [30, 41]. Indeed, there are no specialist tools for cutting of anterior lamina of the transverse process of the cervical vertebrae. Instead, several devices have been adapted for the purpose, with consequences for efficacy, precision, and accuracy of dissection. Other instruments are used to section and remove bone parts of the spine at many levels but are not explicitly aimed at the anterior laminae of the transverse processes of the cervical vertebrae [30]. The use of generic instruments has been noted, such as electric oscillating saws with circumferential or fan blades, surgical scissors (e.g., Metzenbaum, Mayo), forceps, and ossivorous pliers [42, 43], or even rachiotomes by which the spinal cord can be isolated with bloody, rough, and poorly controllable management [9, 44]. These premises necessarily lead to a high probability of iatrogenic injury to vertebral vessels during anatomical dissection for clinical, surgical, medico-legal, or research purposes [20–22]. This, in turn, involves a challenging interpretation of the pathophysiological nature of what has been documented, taking into account anatomical variants [45], pathological entities [46, 47], the consequences of trauma, or a technical error during dissection [48]. The educational, experimental, and cognitive value can be prejudiced and the morphological datum altered [19].

Consequently, a novel dissection tool called the transversoclasiotome was devised and evaluated using anatomical dissections. The composite name “transversoclasiotome” is derived from the Greek -τόμον or -τέμνω, for “cutting,” and -κλᾰ́σῐς, for “rupture,” as the foramen transversarium is opened through cutting and rupture of its anterior lamina. The instrument has been proven suitable for its purpose, comfortable in positioning and maneuvering, and responding adequately to the pressure exerted and resistance felt against the bone lamina. The ergonomic character of the tool was evident, with the operator experiencing no fatigue standing next to the supine-positioned cadaver. The instrument was designed for right-handers and used from the cadaver’s right side, but it can be used on the left side of the neck or applied downward while standing on the left side of the body. The cutting energy could be transferred gradually during the execution of the task. The bone section was cleanly cut and easy to obtain, partially because of the thickness of the anterior lamina of the transverse process (1 mm). The tool did not slide off as it was closed on the bone lamina, acting like a “guillotine” with a wedge effect during the sectioning. As designed, the cutting jaws did not protrude beyond the external/lower profile of the knocker. Hence, the vertebral vessels were not injured during instrument insertion or cutting.

For describing the course of the vertebral artery detected during the anatomical dissections, reference will be made to the individual segments from V1 to V3, omitting segment V4, which is beyond the scope of the present study [49].

The first part (V1—ostial or preforaminal segment) originates at the posterior surface of the ipsilateral subclavian artery, passes anteriorly to the transverse process of C7, and enters the foramen transversarium of the C6 vertebra [50]. This tract is located, together with the vertebral vein positioned ahead of it, in the triangle of the vertebral artery, delimited by the lateral edge of the longus colli muscle, the medial edge of the anterior scalene muscle, and the first part of the subclavian artery [51]. It is located behind the internal jugular vein, here removed for convenience.

The second part (V2—foraminal segment) rises through the foramina transversaria of the cervical vertebrae from C6 to C1, passing from one foramen transversarium to the next and crossing the roots of the cervical plexus in the intertransverse tract anteriorly. The transverse processes from C4 to C6 are located at the same depth in the neck. The C7 transverse process is posterior owing to the cervical lordosis. Consequently, the vertebral artery passes anteriorly at a significant distance from C7 but reaches the transverse process of C6 with no substantial change of direction [52]. The C2 transverse process is inclined downwards with an oblique lateral and inferior orientation, unlike all the other cervical vertebrae, in which it is horizontal and perpendicular to the vertebral body. This implies that the foramen transversarium has an almost sagittal arrangement, so the vertebral artery has to move laterally to reach the C2 transverse process from C3. This is also because the C2 transverse process is longer than the others below it [52]. The anteroposterior diameter of the foramen transversarium decreases from C6 to C3, while the transverse diameter is minimal at C5 [53]. At the foramina transversaria, the vertebral artery is enclosed in a periosteal sheath that is continuous with the one covering the foramina transversaria, within which the vertebral artery is free to slip very little [52] because, at some points, it adheres to the periosteum, from which it must be detached.

Moreover, at the intertransverse tract, the vertebral artery and the roots of the cervical plexus are enclosed by a fibroligamentous band connected to the lateral part of the uncinate process and the related uncovertebral joint, thus forming a single entity [48]. Overall, these anatomical features allow the vertebral artery to be stretched or compressed during movements of the neck without being injured, at the same time guaranteeing blood flow. The mean diameters of the vertebral arteries and their distances from the midline were in line with literature data, and no anatomical variations (fenestration, duplication, or hypoplasia) or abnormal courses were noted in the present case series [54–56].

The third part (V3—suboccipital or extradural or extraspinal segment) extends from the foramen transversarium of C1 to the site of passage through the dura mater. After exiting the foramen transversarium, the vertebral artery curves posteriorly at almost a right angle and then folds medially to engage in the groove for the vertebral artery behind the superior articular facet of the lateral mass of the atlas. In the present case series, there was a standard-shaped groove for the vertebral artery bilaterally, without anterior and posterior osseous bridges arching over the suboccipital segment (V3), transforming the arterial groove into a semi-canal or a complete canal known as the foramen arcuale [57]. This osseous foramen was reported in up to 22% of the general population, housing the vertebral artery, vertebral venous plexus, and suboccipital nerve [57–60]. Its incomplete variant, the semi-canal, was described in up to 28% of the population [57, 59, 60]. However, a meta-analysis showed that the overall incidences of the complete and the semi-canal variants were lower, with prevalences of 9.1% (95% CI 8.2–10.1%) and 13.6% (95% CI 11.2–16.2%), respectively, males predominating in first case and females in the second [61]. Once it has passed over the groove, the vertebral artery folds anteriorly to enter the dura mater behind the occipital condyles, finally ascending through the foramen magnum. The V3 segment can be divided into three parts: a vertical portion that rises through the foramen transversarium of C1, a horizontal portion that flows into the groove for the vertebral artery, and an oblique portion that penetrates the dura mater. This convoluted course is attributable to its passage through mobile bone structures, which can move without injuring the vessel during neck rotation [62].

The fourth part (V4—intradural or intracranial segment) penetrates the dura mater just below the lateral edge of the foramen magnum, creating an invagination of the dura and the periosteal sheath up to 4 mm, with a double coverage enveloping the vertebral artery. Here, the periosteal sheath forms the distal fibrous ring [52]. The fourth part ends at the vertebrobasilar junction.

Along its route, the vertebral artery emits small branches that can be easily preserved through the transversoclasiotome [63]. The spinal branches pass through the intervertebral foramina to enter the spinal canal and supply the spinal cord, its membranes, the vertebral body, and the periosteum. Muscular branches supply the deep muscles at the C1 level. The posterior spinal artery usually originates from the posteromedial surface of the vertebral artery before perforating the dura mater.

The vertebral vein accompanies the vertebral artery along its path. It appears plexiform, progressively converging to a single vessel before connecting with the brachiocephalic vein after it emerges from the foramen transversarium of the C6 vertebra. Occasionally, two vertebral veins have been described, though not in the case series presented [64].

Overall, the transversoclasiotome has been proven appropriate for sectioning the anterior lamina of the transverse process of the cervical vertebrae. It was easy to handle, reacting promptly to the operator’s movement and action and overcoming the bone lamina’s structural resistance to be cut. The tool grasped the bone safely and stably without sliding off during closing. The vertebral vessels were not injured either during the insertion of the instrument or during the bone cutting.

Its use exposed the vertebral vessels for easy examination of their morphological features and anatomical relationships, as required in clinical anatomy for teaching and training clinicians or surgeons; in forensic clinical anatomy during medico-legal ascertainment; and in research.

## Key points

1. The transversoclasiotome is a novel surgical tool.
2. The transversoclasiotome has been proved effective for accessing the foramina transversaria.
3. The tool grasped the bone safely and stably, exposing the vertebral artery easily.
4. It plays a role in dissection for teaching, training, and forensic purposes.
5. Fields of application are clinical anatomy, forensic setting, and research.
